# Umbilical Cord Mesenchymal Stem Cells Combined with Fufang Xueshuantong Capsule Attenuate Oxidative Stress and Vascular Lesions in Diabetic Rats by Activating Nrf-2/HO-1 Signaling Pathway

**DOI:** 10.2174/0118715303251692231112150225

**Published:** 2023-11-22

**Authors:** Yunchao Sun, Yongzhang Li, Xueliang Gao, Limin Gao, Bingqi Yang, Jianing Zhao

**Affiliations:** 1 Department of Vascular Surgery, Hebei Provincial Hospital of Chinese Medicine, Shijiazhuang, China;; 2 Department of Urology, Hebei Provincial Hospital of Chinese Medicine, Shijiazhuang, China;; 3 Department of Neurosurgery, Hebei Provincial Hospital of Chinese Medicine, Shijiazhuang, China;; 4 Department of Conduit Room, Hebei Provincial Hospital of Chinese Medicine, Shijiazhuang, China

**Keywords:** Type 2 diabetes mellitus, umbilical cord mesenchymal stem cells, Fufang Xueshuantong capsule, oxidative stress, vascular lesions, apoptosis

## Abstract

**Background:**

Macrovascular lesions are the main cause of death and disability in diabetes mellitus, and excessive accumulation of cholesterol and lipids can lead to long-term and repeated damage of vascular endothelial cells. Umbilical cord mesenchymal stem cells (UCMSCs) can attenuate vascular endothelial damage in type 1 diabetic mice, while Fufang Xueshuantong capsule (FXC) has a protective effect on endothelial function; however, whether FXC in combination with UCMSCs can improve T2DM macrovascular lesions as well as its mechanism of action are not clear. Therefore, the aim of this study was to reveal the role of FXC + UCMSCs in T2DM vasculopathy and their potential mechanism in the treatment of T2DM.

**Methods:**

The control and T2DM groups were intragastrically administered with equal amounts of saline, the UCMSCs group was injected with UCMSCs (1×10^6^, resuspended cells with 0.5 mL PBS) in the tail vein, the FXC group was intragastrically administered with 0.58 g/kg FXC, and the UCMSCs + FXC group was injected with UCMSCs (1×10^6^) in the tail vein, followed by FXC (0.58 g/kg), for 8 weeks.

**Results:**

We found that FXC+UCMSCs effectively reduced lipid levels (TG, TC, and LDL-C) and ameliorated aortic lesions in T2DM rats. Meanwhile, Nrf2 and HO-1 expression were up-regulated. We demonstrated that inhibition of Nrf-2 expression blocked the inhibitory effect of FXC+UCMSCs-CM on apoptosis and oxidative stress injury.

**Conclusion:**

Our data suggest that FXC+UCMSCs may attenuate oxidative stress injury and macroangiopathy in T2DM by activating the Nrf-2/HO-1 pathway.

## 1 INTRODUCTION

Type 2 diabetes mellitus (T2DM) is a systemic metabolic disease that poses a long-term threat to health and imposes a huge economic burden on patients. It is characterized by insulin resistance, resulting in high blood sugar [1]. Currently, the number of people worldwide with T2DM is increasing exponentially due to various factors, such as an aging population, unhealthy eating habits, and lack of physical activity [2]. Studies have reported that prolonged hyperglycemia can cause a variety of complications of diabetes mellitus, among which macroangiopathy is the most common [3]. In addition, after the glycemia of T2DM is controlled, macroangiopathy may still occur, and it is also a major cause of death and disability in diabetic patients, and about 75%-80% of T2DM patients die from macroangiopathy [4, 5]. Currently, the main treatment strategy for T2DM is to lower blood glucose to minimize the toxicity of hyperglycemia to the organism [6]. However, excessive aggregation of cholesterol, lipids, and inflammatory factors leads to long-term and repeated damage to vascular endothelial cells, which eventually leads to the development of diabetic macroangiopathy [7]. Therefore, it is necessary to explore new therapeutic strategies for T2DM.

Umbilical cord mesenchymal stem cells (UCMSCs) are adult stem cells with multiple differentiation potentials [8]. Previous studies have found that UCMSCs infusion has the effect of improving insulin resistance in T2DM [9]. It can also be used to attenuate vascular endothelial injury in type 1 diabetic mice by inhibiting the hyperglycemia-induced advanced glycosylation end products (AGEs)/receptor for advanced glycosylation end products (RAGE) pathway [10]. Furthermore, practice has proven that traditional Chinese medicine (TCM) is not only effective in the prevention and treatment of diabetes, but also less toxic and with less adverse reactions. As a result, TCM has been widely used in the treatment of diabetes [9]. Fufang Xueshuantong capsule (FXC) is a TCM formula composed of four herbs, including Sanqi (*Panax notoginseng*), Huangqi (*Astragalus membranaceus*), Dansheng (*Salvia miltiorrhiza*), and Xuansheng (*Scrophularia ningpoensis*) [11]. Long-term and extensive clinical applications have confirmed that FXC could exert significant effects on fundus, cardiovascular, and cerebrovascular occlusive diseases [11, 12]. Our previous study found that FXC could effectively increase the serum levels of nitric oxide (NO), endothelial-type nitric oxide synthase (eNOS), and reduce the levels of endothelin-1 (ET-1), which protected the vascular endothelial function in T2DM model rats [13]. However, whether FXC combined with UCMSCs is synergistically effective in T2DM macrovascular lesions has not been investigated.

Nuclear factor NF-E2-related factor 2 (Nrf-2) is a key factor in oxidative stress that binds to an enhancer sequence of the antioxidant response element (ARE) to form the Nrf-2/ARE pathway [14]. The high glucose state produces excessive reactive oxygen species (ROS), which can activate the Nrf-2/ARE pathway and induce a series of protective proteins, such as heme oxygenase 1 (HO-1), to alleviate the damage suffered by the cells, and the activated Nrf-2/HO-1 signaling pathway can play an antioxidant role to promote the healing of diabetic wounds [15]. Furthermore, evidence suggests that Nrf-2 agonists are effective in protecting the organism from diabetic complications [16]. However, Nrf2/HO-1 as an entry point to explore the role of FXC+ UCMSCs in ameliorating T2DM macrovascular lesions has not been demonstrated. To explore the effects and mechanism of FXC combined with UCMSCs to improve macroangiopathy in T2DM, in this study, we observed the effects of FXC combined with UCMSCs on metabolic capacity, histopathological changes, oxidative stress, apoptosis, and Nrf-2/HO-1 signaling pathway in T2DM rats. Moreover, the effects of UCMSCs-CM and FXC-containing serum on cell activity, apoptosis, and oxidative stress, and the effect of inhibiting Nrf-2 expression on the therapeutic effect of UCMSCs-CM combined with FXC-containing serum were also analyzed. Also, the potential regulatory role of FXC combined with UCMSCs in T2DM macroangiopathy has been analyzed, which could be helpful for the treatment of T2DM macroangiopathy.

## 2 MATERIALS AND METHODS

### 2.1 Animals

SPF-grade SD rats (weight 180-220 g) were purchased from Hebei Medical University and housed at 23 ± 2°C and 50 ± 5% humidity. The experiments conducted were in accordance with the guidelines of the Hebei Provincial Laboratory Animal Management Committee. The study was also approved by the Experimental Animal Management Committee of Hebei Medical University (IACUC-Hebmu-p-2021076, date: 2021-04-15).

### 2.2 Experimental Design

Ten rats were randomly selected from fifty SD rats as the control group and were given standard chow (Bio PIKE, China), and the remaining rats were given high-fat chow (with 60 kcal% fat, Bio PIKE, China). All rats were weighed once a week. After eight weeks, the rats fed a high-fat diet were made to fast for 12 h. Then, streptozotocin (STZ, Sigma) 35 mg/kg was injected into the tail vein, and random blood glucose was measured in the tail vein every other day, and blood glucose ≥16.7 mmol/L for 3 consecutive days was considered as successful modeling of T2DM. No rats died during the modeling process, and the high-fat feeding was continued for eight weeks. The rats with successful models were randomly divided into a model group (T2DM, *n*=10), Fufang Xueshuantong capsule group (FXC, *n*=10), umbilical cord mesenchymal stem cells group (UCMSCs, *n*=10), and Fufang Xueshuantong capsule + umbilical cord mesenchymal stem cells group (FXC+ UCMSCs, *n*=10). Then, equal amounts of saline were administered intragastrically to the control and T2DM groups; the UCMSCs group was injected with UCMSCs (1×10^6^ resuspended cells with 0.5 mL PBS, Gibco) in the tail vein, the FXC group was intragastrically administered with 0.58 g/kg FXC (Zhongsheng Pharmacy, China), and the UCMSCs+FXC group was injected with UCMSCs (1×10^6^) in the tail vein, followed by FXC (0.58 g/kg), for 8 weeks. The body weight and blood glucose of rats were monitored weekly. The typical human daily dose of FXC is 4.5 g per 50 kg body weight, and according to the formula: *d*_rat_ = *d*_human_ ×0.71/0.11, the dose of FXC for rat is 0.58 g/kg/day [17].

### 2.3 Sample Collection

Subsequently, sodium pentobarbital was administered by 35 mg/kg intraperitoneal injection to anesthetize rats, blood was taken from the abdominal aorta, and the supernatant was isolated (3000 r/min, 4°C) and stored at -80°C for further analysis. Aortic tissue was taken, and a portion was cut and fixed with 4% paraformaldehyde for pathological observation, while the remaining aorta was stored in the refrigerator at -80°C for molecular detection.

### 2.4 Histopathological Observation

The aortic tissues were placed in 4% paraformaldehyde, fixed and then dehydrated using an alcohol gradient, followed by paraffin-embedded sections (5 μm). The paraffin sections were dewaxed in xylene, covered with alcohol, stained with hematoxylin, fractionated with acetic acid, and then stained with eosin and methylene blue; the fixed amounts were dehydrated and observed under the microscope (Pannoramic 250, Danjier, China). TUNEL staining was used to observe apoptosis in aortic tissue.

### 2.5 Immunofluorescence Staining

For immunofluorescence staining of Nrf-2, aortic tissues were fixed with 4% paraformaldehyde, paraffin-embedded, conventionally sectioned (5 μm), closed with 3% H_2_O_2_ for 10 min, and incubated with Nrf-2 antibody (1:50, Bioss, China) at 4°C overnight, and then secondary antibody (1:1000, GB22303, Servicebio) was added for 30 min at 37°C. Histological examination was performed on a fluorescence scanning microscope (P250 FLASH, Danjier, China), and the relative expression of Nrf-2 was quantified.

### 2.6 Preparation of FXC-containing Serum

20 SPF-grade SD rats weighing 180-220 g were selected, and 10 rats were randomly selected for intragastric administration of FXC at 0.58 g/kg for 5 d. Blood was collected and centrifuged to obtain FXC-containing serum. The other 10 rats were given an equal volume of saline, and the negative control serum was obtained by centrifugation after blood collection.

### 2.7 Preparation of Umbilical Cord Mesenchymal Stem Cells Conditioned Medium

UCMSCs were inoculated into conventional 6-well plates at 2×10^5^/mL, and the culture supernatant was collected after 24 h. The culture supernatant was centrifuged at 1500 r/min for 5 min, filtered through 0.22 μm filter as umbilical cord mesenchymal stem cells conditioned medium (UCMSCs-CM), and stored at -20°C.

### 2.8 Cell Culture

HUVEC cells (Shanghai Tongpai Biotechnology Co., Ltd.) were cultured in a DMEM medium containing 10% fetal bovine serum, 100 U/mL penicillin, 100 μg/mL streptomycin, 40 μU/L insulin, 40 U/mL heparin, and 1% non-essential amino acids. The cultured cells were incubated at 37°C, 5% CO_2_, and saturated humidity. When the cells grew to 70% to 80% fusion, the passages became digested. HUVEC cells at the logarithmic growth stage were inoculated in 6-well culture plates, and after 24 h of inoculation, the cells were treated with different concentrations of FXC-containing serum (0%, 5%, 10%, 15%, 20%, 25%), and the most suitable concentration of FXC serum was selected for the following experiments.

### 2.9 Cell Intervention

Logarithmic growth phase HUVEC cells were inoculated in 6-well culture plates and divided into 6 groups: control group (control), high-sugar group (HS), negative control serum + high sugar group (NC+HS), optimal concentration of serum containing FXC group (FXC), UCMSCs-CM group (UCMSCs-CM), and optimal concentration of serum containing UCMSCs-CM group (FXC+UCMSCs-CM). The control group was given 5.5 mmol/L glucose, the HS group was given 33 mmol/L glucose, the NC+HS group was given 33 mmol/L glucose+25% negative control containing negative control serum concentration, the FXC group was given 33 mmol/L glucose+25% FXC-containing serum concentration, the UCMSCs-CM group was given 33mmol/L glucose and cultured in UCMSCs-CM, and the FXC+UCMSCs-CM group was given 33 mmol/L glucose +FXC (25%) and cultured in UCMSCs-CM.

### 2.10 Cell Transfection

Logarithmic growth phase HUVEC cells were inoculated in 6-well culture plates and divided into 4 groups of si-NC, si-Nrf2-1, si-Nrf2-2, and si-Nrf2-3, for screening of a siRNA with the best interference efficiency using qRT-PCR. Subsequently, the cell groups were set as follows: control group (control), high-sugar group (HS), negative control serum + high-sugar group (NC+HS), optimal concentration of serum containing UCMSCs-CM group (FXC+UCMSCs-CM), FXC+UCMSCs-CM+si-Nrf-2, and FXC+UCMSCs-CM+si-NC group. Lipofectamine 2000 was used according to the manufacturer's instructions. Cells were analyzed 48 hours after transfection.

### 2.11 Biochemical Analysis

Serum levels of high-density lipoprotein cholesterol (HDL-C), low-density lipoprotein cholesterol (LDL-C), triglycerides (TG), and total cholesterol (TC) were measured using the respective kits (NJJCBio, China). In addition, the levels of superoxide dismutase (SOD), malondialdehyde (MDA), and glutathione peroxidase (GSH-Px) in aortic tissues and cells were measured by ELISA with a double antibody sandwich enzyme-linked immunosorbent method (ZCIBIO, Shanghai). All operations were performed in strict accordance with the manufacturer's protocol, and each sample was repeated three times.

### 2.12 Cell Counting Kit-8 Assay

Cells from different treatment groups were incubated at 37°C in 5% CO_2_ for 24 h. Subsequently, the Cell Count Kit-8 (Dojindo Laboratories, Kumamoto, Japan) was used to determine the proliferation of cells according to the operation manual. The absorbance was recorded at 450 nm using a microplate reader (Thermo Fisher Scientific, Waltham, MA, USA).

### 2.13 Flow Cytometry

Cells from different treatment groups were incubated at 37°C in 5% CO_2_ for 24 h. Subsequently, cell apoptosis and ROS content were detected by flow cytometry kit (Sigma Aldrich), respectively.

### 2.14 Reverse Transcription-Quantitative Polymerase Chain Reaction (RT-qPCR)

The total RNA was extracted from cells using the TRIzol kit (Invitrogen) according to the manufacturer's instructions. Total RNA was taken and reverse transcribed to synthesize cDNA. Gene expression levels were quantified using TB Green^TM^ Premix Ex Taq^TM^ II (Tli RNaseH Plus) with β-actin as an internal reference. The PCR amplification reaction conditions were as follows: 95°C pre-denaturation for 10 min, 95°C denaturation for 15 s, and 60°C annealing extension for 45 s, all carried out for 40 cycles. Primer sequences (Sangon, Shanghai) used are as follows: Nrf-2: forward primer (5’-3’) GCTGTGCCTATGTCTCAGCCTCTTCT, reverse primer (5’-3’) GGTGGTTTGTGAGTGTGAGGGTCT GG; β-actin: forward primer (5’-3’) GAAGATCAAGATC ATTGCTCC, reverse primer (5’-3’) TACTCCTGCTTGCT GATCCA. The formula Q= 2^-∆∆CT^ was used to calculate the relative expression of mRNA.

### 2.15 Western Blot Analysis

Proteins were extracted from cells using radioimmuno-precipitation assay buffer (RIPA) (Servicebio, China). The protein concentrations were then quantified using the BCA protein assay kit (Beyotime, China) and subjected to SDS-PAGE electrophoresis. The anti-Nrf-2 antibody (ab137550) and anti-HO-1 antibody (ab223349) obtained from Abcam (UK) were utilized for immune reaction. Finally, a chemiluminescence detection system was used to detect the samples, and ImageJ software (Image-J, National Institutes of Health, USA) was used to quantify the intensity of the bands; PARP or β-actin was used as an internal reference.

### 2.16 Statistical Analysis

Data were statistically analyzed using SPSS 19.0 software (SPSS Inc., Chicago, USA), and the Shapiro-Wilk test was used to verify whether the data conformed to normal distribution, and the measurements that conformed to normal distribution were expressed as the mean ± standard deviation (mean±SD). One-way ANOVA was used for between-group comparisons, and a logarithmic transformation was performed when the data distribution did not conform to normal distribution, and the transformed data were subjected to one-way ANOVA. Among the data, when the variance was homogeneous, the LSD-*t* test was used for two-to-two comparisons between the groups, and when the variance was not homogeneous, Tamhane's T2 test was used for two-to-two comparisons between the groups. *p* <0.05 was considered as a statistically significant difference.

## 3 RESULTS

### 3.1 Effect of FXC Combined with UCMSCs on the Metabolism of Diabetic Rats

**Fig. (1):**
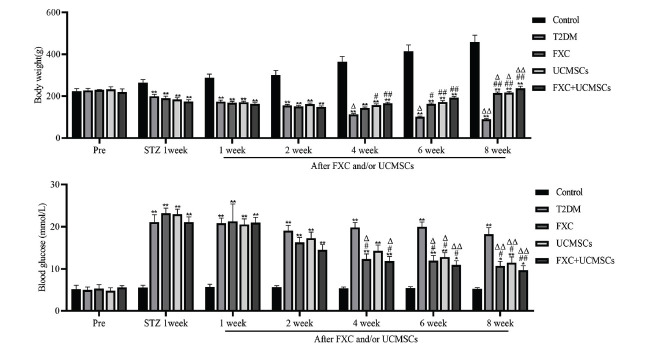
Effect of FXC combined with UCMSCs on the metabolism of diabetic rats; changes in body weight and blood glucose in each group of rats. Data are shown as mean ± SD. *p <0.05, **p <0.01 compared to the control group; ^#^*p* <0.05, ^##^*p* <0.01 compared to the T2DM group; ^Δ^*p* <0.05, ^ΔΔ^*p* <0.01 compared to 1 week.

Long-term abnormalities of glucose-lipid metabolism in diabetes cause pathological changes in the kidney, and diabetic nephropathy is one of the most common and serious microvascular complications of diabetes. Firstly, the effects of FXC in combination with UCMSCs on the metabolism of diabetic rats were investigated. The results showed the body weight of rats in the FXC, the UCMSCs, and the FXC+UCMSCs groups to be significantly increased at 6 and 8 weeks compared to the T2DM group (*p* <0.05, Fig. **1**), and blood glucose was also observably reduced at 6 and 8 weeks in the FXC, the UCMSCs, and the FXC+UCMSCs groups of rats (*p* <0.05, Fig. **1**). Compared to 1 week after administration, the T2DM group showed a gradual decrease in body weight over time, whereas the FXC, the UCMSCs, and the FXC+UCMSCs groups showed a significant increase in body weight and a significant decrease in blood glucose after 8 weeks of administration (*p* <0.05, Fig. **1**). In addition, the levels of TG, TC, and LDL-C were markedly lower (*p* <0.05, Table **1**) in the UCMSCs and FXC+UCMSCs groups compared to the T2DM group, indicating that the metabolic capacity of rats with T2DM was weaker than that of the control group. However, FXC, UCMSCs, and FXC+UCMSCs had a significant effect on the metabolic capacity of T2DM rats.

### 3.2 Effect of FXC Combined with UCMSCs on the Aorta of Diabetic Rats

**Fig. (2):**
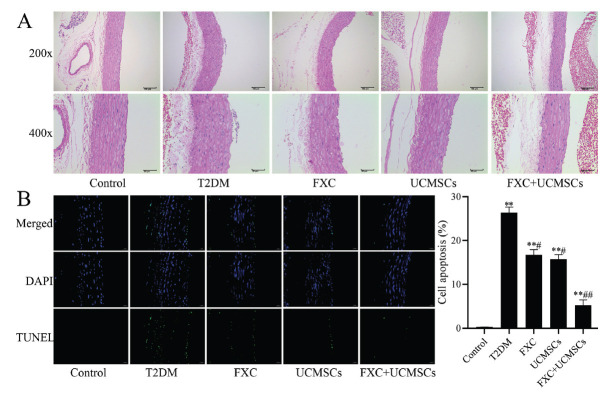
Effect of FXC combined with UCMSCs on the aorta of diabetic rats. (**A**) Aortic tissue was observed by HE staining, magnification: ×200 and ×400; (**B**) Apoptosis in aortic tissue was observed by TUNEL staining, magnification: ×400. Data are shown as mean ± SD. **p <0.01 compared to the control group; ^#^*p* <0.05, ^##^*p* <0.01 compared to the T2DM group.

Hyperglycemia promotes vascular complications, the pathology of which often involves blood vessels; thus, we observed the effects of FXC combined with UCMSCs on the aorta of diabetic rats; histopathological and apoptotic analyses of the rats were performed. The results found that compared to the control group, a large number of endothelial cells were swollen and shed in aortic tissue (Fig. **2A**), and apoptosis was also significantly increased in aortic tissue (*p* <0.01, Fig. **2B**) of T2DM rats. However, a small number of endothelial cells in aortic tissue were slightly swollen, and apoptosis was significantly reduced in the FXC, UCMSCs, and FXC+UCMSCs groups than in the T2DM group (*p* <0.05), demonstrating that FXC, UCMSCs, and FXC+ UCMSCs treatments were able to protect the aorta of T2DM rats.

### 3.3 Effect of FXC Combined with UCMSCs on Oxidative Stress in Diabetic Rats

**Fig. (3):**
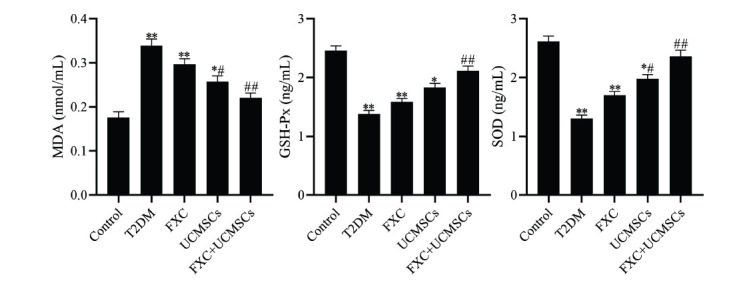
Effect of FXC combined with UCMSCs on oxidative stress in diabetic rats; the levels of MDA, GSH-Px, and SOD in aortic tissue. Data are shown as mean ± SD. *p <0.05, **p <0.01 compared to the control group, ^#^*p* <0.05, ^##^*p* <0.01 compared to the T2DM group.

Effective inhibition of oxidative stress in diabetic patients attenuates diabetes; to further verify the effect of FXC combined with UCMSCs on oxidative stress in diabetic rats, relevant parameters were investigated by ELISA. The present results displayed the level of MDA in the aortic tissue to be increased and the levels of SOD and GSH-Px as decreased in the T2DM group compared to the control group (*p* <0.01). Compared to the T2DM group, MDA levels were markedly decreased, and the levels of SOD and GSH-Px were dramatically increased in the aortic tissue of rats in the FXC+UCMSCs group (*p* <0.01). It was observed that FXC+UCMSCs attenuated oxidative stress damage caused by T2DM (Fig. **3**).

### 3.4 Effects of FXC Combined with UCMSCs on Nrf-2/HO-1 Signaling Pathway and Apoptosis-related Protein Expression in Diabetic Rats

**Fig. (4):**
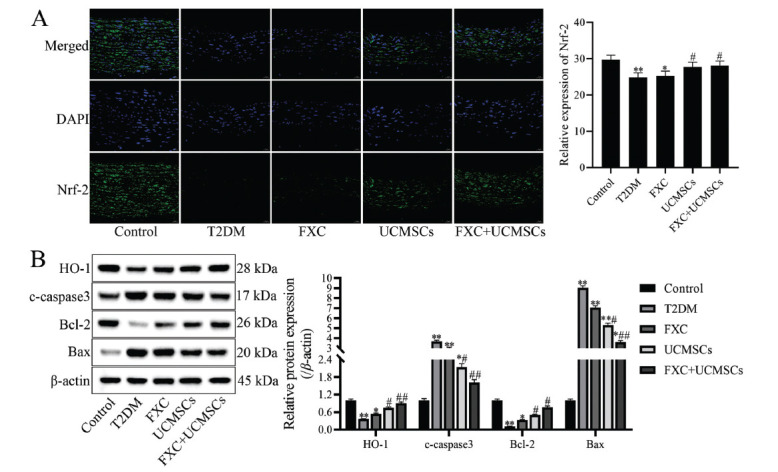
Effects of FXC combined with UCMSCs on Nrf-2/HO-1 signaling pathway and apoptosis-related protein expression in diabetic rats. (**A**) The expression of Nrf-2 in aortic tissue was detected by immunofluorescence, magnification: ×40; (**B**) The protein expression of HO-1, cleaved caspase-3, Bcl-2, and Bax in aortic tissue was detected by western blot, and densitometry analysis of the intensity of the protein bands was performed. Data are shown as mean ± SD. *p <0.05, **p <0.01 compared to the control group; ^#^*p* <0.05, ^##^*p* <0.01 compared to the T2DM group.

Nrf-2/HO-1 signaling pathway is closely related to oxidative stress; in this regard, we used an immunofluorescence assay to detect the expression of Nrf-2 in rat aortic tissues, and the protein expressions of HO-1, cleaved caspase-3, Bcl-2, and Bax in aortic tissue were detected by Western blot. The results showed the expressions of Nrf-2, HO-1, and Bcl-2 to be significantly decreased in aortic tissue, and the expressions of cleaved caspase-3 and Bax were markedly increased in aortic tissue of rats in the T2DM group compared to the control group (*p* <0.01). In contrast, the expressions of Nrf-2, HO-1, and Bcl-2 were dramatically increased and those of cleaved caspase-3 and Bax were significantly reduced in aortic tissue in the UCMSCs and FXC+UCMSCs groups (*p* <0.05), indicating that UCMSCs and FXC+ UCMSCs could affect the Nrf-2/HO-1 signaling pathway and inhibit pro-apoptotic protein expression in T2DM rats (Fig. **4**).

### 3.5 Effects of UCMSCs-CM Combined with FXC-containing Serum on Cell Activity, Apoptosis, and Oxidative Stress

**Fig. (5):**
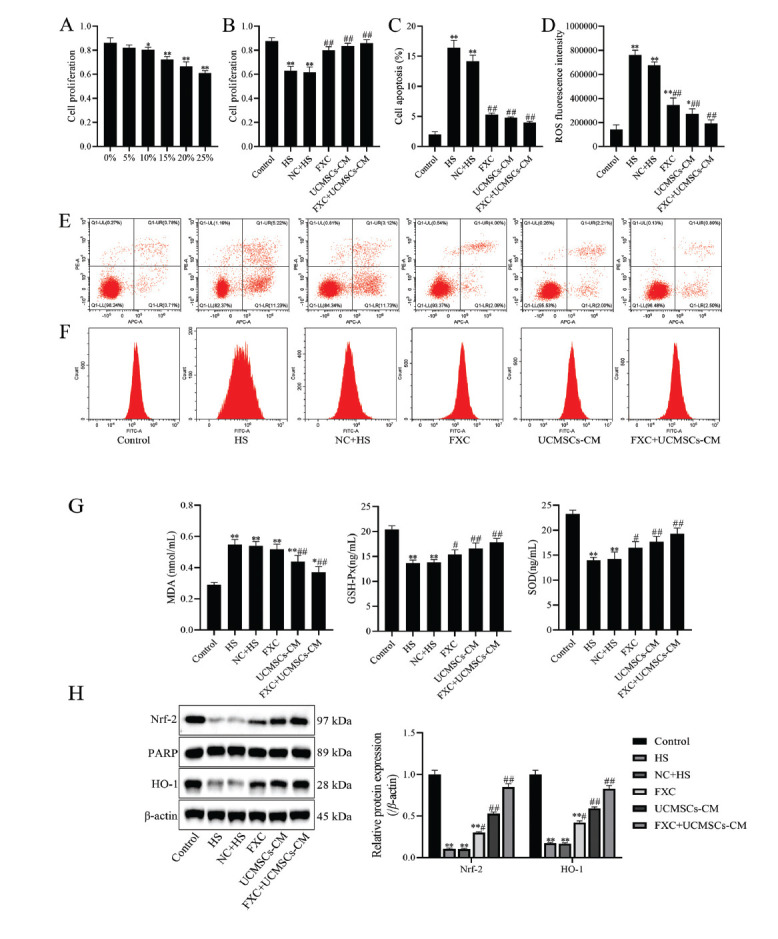
Effects of UCMSCs-CM combined with FXC-containing serum on cell activity, apoptotic, and oxidative stress. (**A**) The effect of different concentrations of FXC serum on cell proliferation was measured by CCK-8; (**B**) Cell proliferation was measured by CCK-8; (**C**) Cell apoptosis was analyzed by flow cytometry; (**D**) ROS fluorescence intensity was analyzed by flow cytometry; (**E**) Apoptosis was determined by flow cytometry; (**F**) ROS was assessed by flow cytometry; (**G**) The levels of MDA, GSH-Px, and SOD were measured by ELISA; (**H**) The protein expressions of Nrf-2 and HO-1 were detected by western blot, and densitometry analysis of the intensity of protein bands was performed. Data are shown as mean ± SD. *p <0.05, **p <0.01 compared to the control group; ^#^*p* <0.05, ^##^*p* <0.01 compared to the HS group.

Further, *in vitro* experiments were performed to observe the effects of FXC in combination with UCMSCs; firstly, HUVEC cell activity was detected by CCK-8 to screen the optimal serum concentration of FXC, and the changes in cell activity, apoptosis, oxidative stress, and Nrf-2/HO-1 signaling pathway were detected by CCK-8, flow cytometry, ELISA, and western blot. The results showed that the proliferation of the HUVEC cells group containing 25% FXC serum was significantly reduced. Compared to the control group, cell proliferation was significantly reduced in the HS group, while cell proliferation was significantly increased in the FXC, UCMSCs-CM, and FXC+UCMSCs-CM groups (*p* <0.01, Fig. **5A** and **B**). Compared to the control group, apoptosis and ROS content were significantly increased in the HS group, while apoptosis and ROS content were significantly decreased in the FXC, UCMSCs-CM, and FXC+UCMSCs-CM groups (*p* <0.01, Fig. **5C**-**F**). The levels of MDA were significantly increased, and the levels of SOD and GSH-Px were significantly decreased in the HS group compared to the control group (*p* <0.01). Compared to the HS group, the levels of MDA were markedly decreased, and the levels of SOD and GSH-Px were dramatically increased in the UCMSCs-CM and FXC+UCMSCs-CM groups (*p* <0.01, Fig. **5G**). In addition, the protein expression of Nrf-2 and HO-1 was significantly reduced in the HS group (*p* <0.05), while the protein expression of Nrf-2 and HO-1 was significantly increased in the FXC, UCMSCs-CM, and FXC+UCMSCs-CM groups (*p* <0.05, Fig. **5H**), suggesting that UCMSCs-CM and FXC+UCMSCs-CM inhibited apoptosis and promoted activation of the Nrf-2/HO-1 signaling pathway.

### 3.6 Effect of Nrf-2 Inhibition on the UCMSCs-CM Combined with FXC-containing SERUM

**Fig. (6):**
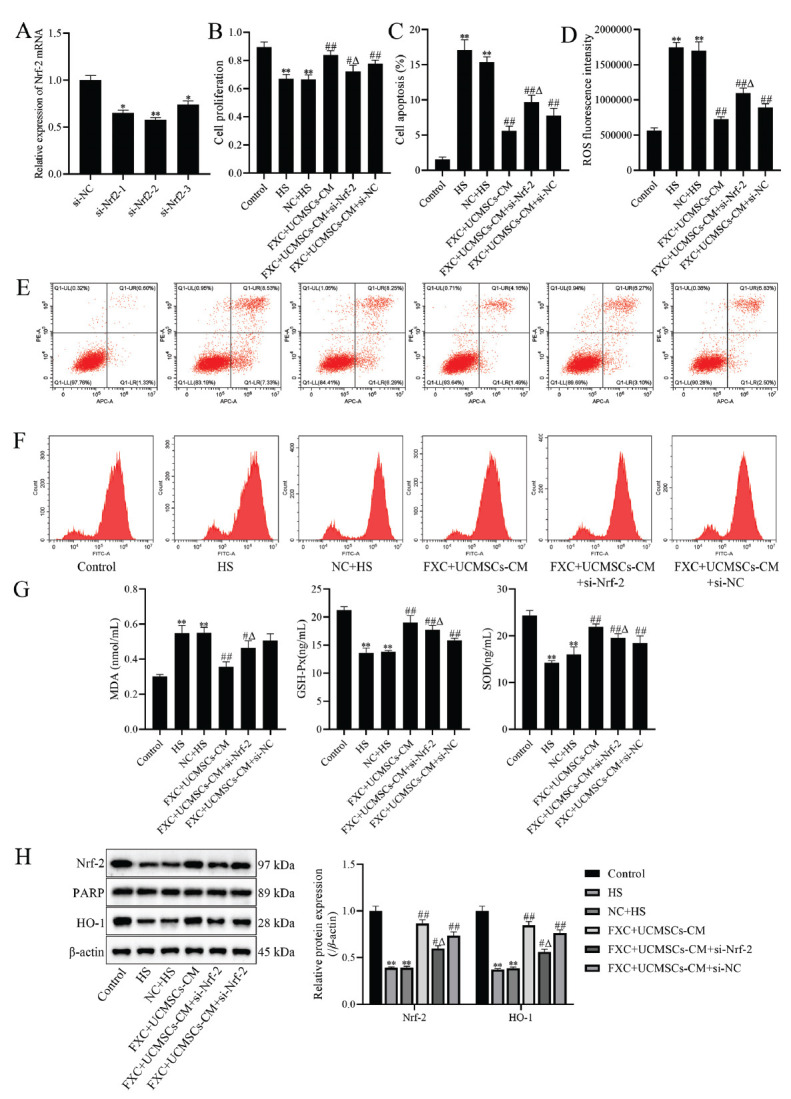
Effect of Nrf-2 inhibition on the effect of UCMSCs-CM combined with FXC-containing serum. (**A**) The mRNA expression of Nrf-2 was measured by qRT-PCR; (**B**) Cell proliferation was measured by CCK-8; (**C**) Cell apoptosis was analyzed by flow cytometry; (**D**) ROS fluorescence intensity was analyzed by flow cytometry; (**E**) Apoptosis was determined by flow cytometry; (**F**) ROS was evaluated by flow cytometry; (**G**) The levels of MDA, GSH-Px, and SOD were measured by ELISA; (**H**) The protein expression of Nrf-2 and HO-1 was detected by western blot, and densitometry analysis of the intensity of the protein bands was performed. Data are shown as mean ± SD. *p <0.05, **p <0.01 compared to si-NC or control group; ^#^*p* <0.05, ^##^*p* <0.01 compared to the HS group; ^Δ^*p* <0.05 compared to FXC+UCMSCs-CM group.

To confirm the effect of inhibiting Nrf-2 expression on UCMSCs-CM in combination with FXC-containing serum, we examined the effects of FXC+UCMSCs-CM on cell proliferation, apoptosis, and oxidative stress after transfection with si-Nrf-2. The results revealed the mRNA expression of Nrf-2 to be significantly reduced after transfection with si-Nrf-2 (*p* <0.05, Fig. **6A**). Compared to the HS group, cell proliferation was significantly increased, and apoptosis and ROS content were significantly decreased in the FXC+UCM SCs-CM, FXC+UCMSCs-CM+si-Nrf-2, and FXC+UCM SCs-CM+si-NC groups; however, si-Nrf-2 transfection significantly inhibited the effects of FXC+UCMSCs-CM on cell proliferation promotion and apoptosis inhibition (*p* <0.05, Fig. **6B**-**F**). Compared to the HS group, the levels of MDA were markedly decreased, and the levels of SOD and GSH-Px were dramatically increased in the FXC+UCMSCs-CM and FXC+UCMSCs-CM+si-Nrf-2 groups, while transfection with si-Nrf-2 significantly inhibited this effect (*p* <0.05, Fig. **6G**). Moreover, the protein expression of Nrf-2 and HO-1 was significantly increased in the FXC+UCMSCs-CM, FXC+UCMSCs-CM+si-Nrf-2, and FXC +UCMSCs-CM+si-NC groups, compared to the HS group, while transfection with si-Nrf-2 significantly inhibited this effect (*p* <0.05, Fig. **6H**), suggesting that inhibition of Nrf-2 expression significantly attenuated the inhibitory effect of FXC+UCMSCs-CM on apoptosis and oxidative stress damage.

## 4 DISCUSSION

In the present study, FXC+UCMSCs have been found to ameliorate aortic pathology and oxidative stress in T2DM rats and modulate the Nrf-2/HO-1 signaling pathway. The results revealed that inhibition of Nrf-2 expression significantly suppressed the inhibitory effects of FXC+ UCMSCs-CM on apoptosis and oxidative stress injury. These findings confirmed that FXC+UCMSCs may exert an ameliorative effect on T2DM by affecting apoptosis and oxidative stress through the Nrf-2/HO-1 signaling pathway.

Diabetes mellitus (DM) is a metabolic disease characterized by chronic hyperglycemia caused by a variety of etiologies [18]. Patients with DM have disorders of glucolipid metabolism, with elevated levels of TC, TG, and LDL in the blood and decreased concentrations of HDL, which plays a protective role [19]. In addition, studies have confirmed that long-term abnormalities of glucolipid metabolism in T2DM cause pathological changes in the aorta [20]. It has been shown that apoptosis and the occurrence of aortic injury in T2DM are closely related [21]. In this experiment, the blood glucose, TG, TC, and LDL-C levels were significantly increased in the T2DM group of rats, T2DM caused aortic injury, and the expressions of apoptosis and apoptosis-related proteins, cleaved caspase-3 and Bax, were significantly increased in the aortic tissues of rats. The treatment of FXC+UCMSCs was able to attenuate the aortic injury of T2DM, and significantly inhibited the blood glucose, TG, TC, LDL-C, cleaved caspase-3, and Bax levels in rats. These results suggest that FXC+UCMSCs can ameliorate the occurrence of dyslipidemia and apoptosis in T2DM rats.

Oxidative stress plays an extremely important role in the development of T2DM, and it has been shown that diabetic states are characterized by increased MDA levels, decreased SOD activity, and elevated oxidative stress [22, 23]. Effective inhibition of oxidative stress in diabetic patients can alleviate diabetes; for *e.g.*, Liao *et al*. [24] reported that polysaccharides from Okra exerted a protective effect on T2DM mice by altering oxidative stress. Furthermore, impairment of vascular endothelial cell function is the pathological basis of vascular injury in T2DM, and oxidative stress is involved in all aspects of the pathophysiological process; therefore, mitigation of oxidative stress is an important approach to protect vascular endothelial cells [25, 26]. This is consistent with the results of the present study, which showed MDA content to be significantly increased, and SOD and GSH-Px activities to be significantly weakened in T2DM rats. FXC+UCMSCs were able to significantly reduce MDA content and enhance SOD and GSH-Px activities in T2DM rats, suggesting that FXC+UCMSCs could inhibit oxidative stress in T2DM rats and thus alleviate diabetes. In addition, the antioxidant effect of FXC+UCMSCs was further verified in this experiment using cellular assay, and the results showed that FXC+UCMSCs-CM was able to reduce the MDA level and increase the activities of SOD and GSH-Px in the cellular supernatant, which further indicated that the protective effect of FXC+UCMSCs on T2DM was realized through antioxidant effect.

The Nrf-2/HO-1 signaling pathway is closely related to oxidative stress, and studies have shown that compounds that upregulate Nrf-2 are effective in protecting the body from diabetic complications [27, 28]. Nrf-2 is also considered to be the most sensitive signal against oxidative stress [29]. Mittal *et al.* [30] demonstrated that activation of the Nrf-2/HO-1 signaling pathway reduced renal insufficiency and oxidative stress levels in diabetic mice. Baig *et al.* [31] reported that the anti-nephropathic effect of C. anthelminticum can be attributed to its ability to down-regulate NF-κB and bring the expression of Nrf-2 to near-normal levels. In the present study, the expressions of Nrf-2 and HO-1 were decreased in T2DM rats, and FXC+UCMSCs were able to activate the Nrf-2/HO-1 signaling pathway. *In vitro* cellular experiments have demonstrated consistent results that FXC+UCMSCs-CM increased the activation of the Nrf-2/HO-1 signaling pathway in high glucose-induced HUVEC cells. We further employed si-Nrf-2 to inhibit the activation of the Nrf-2/HO-1 signaling pathway in cells to validate the role of the Nrf-2/HO-1 signaling pathway in the treatment of FXC+UCMSCs, and the results showed that the inhibition of Nrf-2 expression attenuated the effects of FXC+UCMSCs-CM on promoting cell proliferation, inducing apoptosis, and resisting oxidative stress. These results suggest that FXC+UCMSCs may play a role in ameliorating T2DM macroangiopathy by regulating the Nrf-2/HO-1 signaling pathway against T2DM oxidative stress.

## CONCLUSION

In conclusion, we have clarified that FXC+UCMSCs attenuated oxidative stress injury and macroangiopathy, and activated the Nrf-2/HO-1 signaling pathway in T2DM. These results suggest that FXC+UCMSCs may improve T2DM directly or indirectly by regulating the Nrf-2/HO-1 signaling pathway. In short, we hope that our findings will provide a solid evidence base for the treatment of diabetic vasculopathy.

## FUTURE PERSPECTIVE

There are a few limitations in the present study. The effect of activation of the Nrf2/HO-1 pathway by FXC+UCMSCs on oxidative stress and aortic injury in T2DM rats was not investigated in this study using an animal model. As a next step, our group will conduct *in vivo* validation experiments using Nrf2 inhibitors; moreover, this study was conducted on experimental animals and cells, so continued clinical studies are needed in the future.

## Figures and Tables

**Table 1:** Blood lipid of each group of rats (*n*=10).

**Groups**	**TG (mmol/L)**	**TC (mmol/L)**	**HDL-C (mmol/L)**	**LDL-C (mmol/L)**
Control	0.88±0.02	2.19±0.25	1.70±0.29	0.33±0.09
T2DM	7.91±1.31**	3.91±0.56**	0.49±0.09**	2.98±0.76**
FXC	5.99±1.50**	2.74±0.24^##^	0.59±0.16**	1.83±0.20^**#^
UCMSCs	4.35±1.23^**##^	2.38±0.37^##^	0.71±0.06**	1.09±0.37^##^
FXC+UCMSCs	1.57±0.51^##^	2.25±0.13^##^	1.08±0.15^**##^	0.95±0.25^##^

**Note:** Data are shown as mean ± SD. **p <0.01 compared to the control group; ^#^*p* <0.05, ^##^*p* <0.01 compared to the T2DM group.

## Data Availability

The data and supportive information are available within the article.

## LIST OF ABBREVIATIONS

AGEs: Advanced Glycosylation End Products
FXC: Fufang Xueshuantong Capsule
NO: Nitric Oxide
RAGE: Receptor for Advanced Glycosylation End Products
T2DM: Type 2 Diabetes Mellitus
UCMSCs: Umbilical Cord Mesenchymal Stem Cells
